# MIGRATION, MOBILITY, AND PURSUIT OF GOOD OLD AGES: NARRATIVES OF OLDER PUERTO RICAN ADULTS WHO MIGRATE

**DOI:** 10.1093/geroni/igz038.2616

**Published:** 2019-11-08

**Authors:** Brooke V Jespersen

**Affiliations:** Case Western Reserve University, Cleveland, Ohio, United States

## Abstract

Migration research has articulated “regimes of mobility,” or multi-scalar movements (within and across households, communities, and borders) that are interconnected and embedded in unequal power relations. Research on late-life migration has been limited by: (1) simplistic conceptualizations of mobility as adaptive or traumatic; and (2) a focus on transnationalism. The migration of older adults between Puerto Rico and US mainland presents a new frontier for examining mobility in aging. Puerto Rico’s population is rapidly aging and out-migrating. Moreover, as US citizens, Puerto Ricans experience no legal restrictions on migration typical of transnationalism. Yet little is known about their migration patterns and associated narrated meanings. I conducted semi-structured interviews and participant observation among older Puerto Ricans who migrated to the US mainland in late-life. Preliminary findings suggest that older Puerto Ricans negotiate competing definitions of “good” old ages based on residential context. They report migrating to the mainland to pursue “good” old ages defined in material terms, namely access to social and medical services. Post-migration, however, older Puerto Ricans report experiences of confinement and loneliness, due to language barriers and familial separation. In narrating hopes for the future, they describe an alternative “good” old age in Puerto Rico, emphasizing belonging and familial connection. As older Puerto Ricans negotiate multiple definitions of “good” old ages through circular mobility, the social and economic inequalities which first necessitated migration reproduce disadvantage in the new location. This study highlights the need to conceptualize multi-scalar mobilities that intersect with inequality to shape aging among migrant populations.

